# Multi-TransDTI: Transformer for Drug–Target Interaction Prediction Based on Simple Universal Dictionaries with Multi-View Strategy

**DOI:** 10.3390/biom12050644

**Published:** 2022-04-27

**Authors:** Gan Wang, Xudong Zhang, Zheng Pan, Alfonso Rodríguez Patón, Shuang Wang, Tao Song, Yuanqiang Gu

**Affiliations:** 1 College of Computer Science and Technology, China University of Petroleum, Qingdao 266580, China; nick@s.upc.edu.cn (G.W.); east@s.upc.edu.cn (X.Z.); wangshuang@upc.edu.cn (S.W.); 2 Department of Accounting and Information Systems, University of Canterbury, Christchurch 8041, New Zealand; pan.zheng@canterbury.ac.nz; 3 Department of Artificial Intelligence, Faculty of Computer Science, Polytechnical University of Madrid, Campus de Montegancedo, 28660 Madrid, Spain; arpaton@fi.upm.es; 4 Qingdao Health Talents Development Center, Qingdao 266003, China; qdwsrc@163.com

**Keywords:** DTI prediction, deep learning, transformer, multi-view strategy, embedding dictionary

## Abstract

Prediction on drug–target interaction has always been a crucial link for drug discovery and repositioning, which have witnessed tremendous progress in recent years. Despite many efforts made, the existing representation learning or feature generation approaches of both drugs and proteins remain complicated as well as in high dimension. In addition, it is difficult for current methods to extract local important residues from sequence information while remaining focused on global structure. At the same time, massive data is not always easily accessible, which makes model learning from small datasets imminent. As a result, we propose an end-to-end learning model with SUPD and SUDD methods to encode drugs and proteins, which not only leave out the complicated feature extraction process but also greatly reduce the dimension of the embedding matrix. Meanwhile, we use a multi-view strategy with a transformer to extract local important residues of proteins for better representation learning. Finally, we evaluate our model on the BindingDB dataset in comparisons with different state-of-the-art models from comprehensive indicators. In results of 100% BindingDB, our AUC, AUPR, ACC, and F1-score reached 90.9%, 89.8%, 84.2%, and 84.3% respectively, which successively exceed the average values of other models by 2.2%, 2.3%, 2.6%, and 2.6%. Moreover, our model also generally surpasses their performance on 30% and 50% BindingDB datasets.

## 1. Introduction 

**Figure 1:**
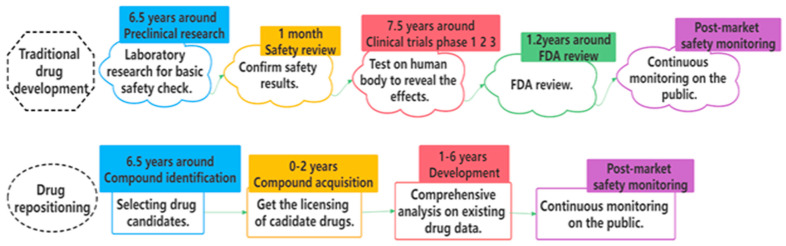
Two flowcharts on comparisons between traditional drug development and drug repositioning.

With the intense struggle between COVID-19 and mankind, there has been a growing number of investments and attention towards drug repositioning. Drug discovery is a time-consuming, expensive, and laborious process full of ups and downs [1]. It generally takes more than 10 years to develop a new drug, with a success rate of only 2.01% [2,3]. Traditional drug development mainly consists of five stages [2]: preclinical research, safety review, clinical trials, FDA review, and post-market safety monitoring. By comparison, drug repositioning provides more effective ways and alleviates the bottlenecks of time and cost for many countries. Figure 1 displays the whole comparison process [2]. In drug repositioning, the identification of potential drug–target interactions (DTIs) plays an extraordinary role in the early stage of drug development [4,5]. Luckily, with the accumulation of more targets, drugs, and their interaction data, various computational approaches have been developed and become accessible in recent years [6]. It is rather promising and valuable to extract and refine their merits to realize unique contributions of knowledge and accelerate the process of drug repositioning. Therefore, we propose Multi-TransDTI for more effective predictions of potential drug–target interactions.

The computational methods for DTIs can be roughly categorized into the following four relatively superior and advanced methods, on the basis of theoretical and principal differences [7,8]: Firstly, matrix factorization has been implemented in DTIs tasks for a long time. Gonen [9] implemented Bayesian matrix factorization with twin kernels and Ezzat [10] used regularized matrix factorization methods to approximately multiply matrices representing the drug and target to obtain an interaction matrix and similarity score matrix; this method demonstrated the highest performance in comparison with previous methods at the time. Secondly, studies have focused on several docking methods which simulated the binding site between a molecule and a protein in a 3D structure. With the emergence of a series of open-source 3D docking programs such as AutoDock [11] and Smina [12], F. Wan [13] conducted molecular docking studies for DTIs using AutoDock Vina [11] on DUD-E, which is a benchmark dataset widely used for evaluating molecular docking. H. Li [14] also proposed a docking method based on a random forest to improve predictive performance. Thirdly, machine learning-based prediction methods have also flourished in multiple aspects [15], maintaining relatively superior merits. Optimized models based on machine learning, such as support vector machines (SVM) [16], Gaussian interaction profiles (GIP) [17], random forest (RM) [18], random walk with restart (RWR) [19,20], and so on, have been implemented to effectively identify potential compound–protein interactions (CPIs). Despite the promising advances and prospects proposed in above methods, they still face some major obstacles. Most of the docking and machine learning-based methods rely on the assumption of structural similarity between different biological entities [21,22]; requiring a vast amount of domain knowledge makes it difficult to obtain the 3D structure of entities [23], particularly when it comes to large-scale datasets. In addition, researchers have found that the similarity of protein sequences sharing an identical interacting drug is not strongly correlated [21,24]. Moreover, similarity-based methods work well for DTIs within specific target classes but not others. In addition, the matrix factorization method has been shown to be not generalizable to different target classes [5,22]. Luckily, another final category is the deep learning-based approach, which has risen rapidly in recent years and revolutionized DTIs. 

Just as investment, consumption, and export are the major forces driving the economy, the deep learning-based approaches driving DTI performance have their characteristics as well. Differences among various deep learning methods mainly lie in data preprocessing, model architecture, and learning strategy. Lee [21] simply used a deep network for drug fingerprints and CNN for proteins to obtain prediction outcomes. J. Peng [25] implemented a restart random walk to extract features from the heterogeneous network and convolutional neural network to complete DTI predictions. F. Wan [13] constructed corpora for generating both protein and drug feature vectors and then flowed them to multimodal neural networks to obtain binding scores. Both B. Y. Ji [26] and [27] proposed a novel network embedding for data preprocessing. K. Abbasi [28] combined CNN [29,30] and LSTM to encode compounds and proteins for predicting DTIs. In recent years, with the breakthrough of transformer models in the field of natural language processing, transformer-based methods such as those described by L. Chen [31] and K. Huang [5] have been developed to further improve representation learning for DTIs. Although progress has been made in potential innovations, the following limitations remain: Current representation learning or feature generation approaches of both proteins and drugs are rather complicated. Moreover, current embedding methods such as that described by I. Lee [21] do not take into account the relationship between each character in the sequence, which often results in a high-dimensional embedding matrix and produces partial data noise. In addition, it is still challenging to effectively obtain important local residues while focusing on global structure. As a result, we propose an end-to-end learning model with a multi-view strategy based on Simple Universal protein and drug dictionaries (SUPD and SUDD) for better embedding [21,32,33], namely Multi-TransDTI, which fully takes into consideration the concerns discussed above.

## 2. Materials and Methods

### 2.1. Our Datasets

The production of our BindingDB dataset can be divided into three main steps. Given that the original BindingDB dataset [5,34] was unbalanced, where the proportion of positive and negative samples was approximately 1:3, it is difficult for us to evaluate the performance of the model from multiple perspectives. Firstly, we downloaded the original BingdingDB dataset from Moltrans [5]. Secondly, we processed the dataset to obtain a balanced one with zero duplicate samples. Thirdly, we divided the dataset into training, validation, and test sets.

In the first step, we obtained 9166 positive samples and 23,435 negative samples, where positive samples have a label of 1 between drugs and proteins, indicating the interaction between them. The original BindingDB dataset in Moltrans provided data for 10,665 drugs and 1413 proteins [5]. After removing 6256 duplicate samples, we were left with 26,336 non-duplicate samples: 6575 positive ones and 19761 negative ones. 

In the second step, we removed four positive samples because the drugs involved could not generate their corresponding Morgan fingerprints as a binary vector indicating the existence of specific substructures [21,35]. This left us with 6571 positive samples. In order to obtain a balanced dataset, we randomly selected 6571 negative samples from all negative samples. Ultimately, we included 6571 positive samples, 6571 negative samples, 7137 drugs, and 1253 proteins. The specific details of the newly BindingDB dataset are shown in Table 1.

In the third step, we divided all samples into training, validation, and test sets according to the ratio of 7:1.5:1.5. Ultimately, we included 9200 samples in the training set, 1970 samples in the validation set, and 1972 samples in the test set. The proportion of positive and negative samples in each set is 1:1. Meanwhile, we randomly selected 50% and 30% of the training set to construct 50% and 30% datasets for evaluating our model on small datasets. The specific information is shown in Table 2. All models were only trained by the training sets; then, the optimal model among them was identified through validation sets. Ultimately, all experimental results were obtained by applying the selected models to the test sets.

**Figure 2:**
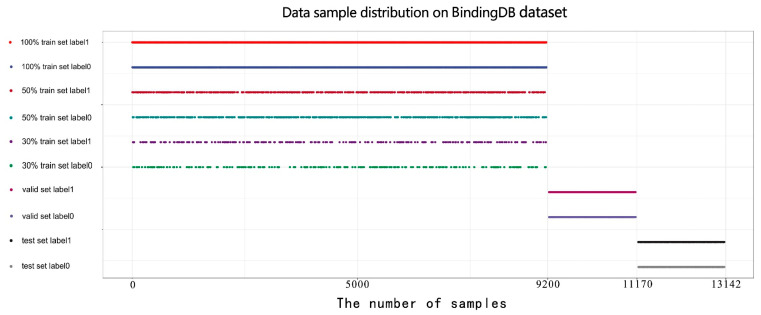
Data sample distribution on our customized BindingDB dataset.

The sample data distribution is shown below in Figure 2, which clearly shows that the validation and test data are completely new and have not appeared in the training set. The proportion of positive and negative samples in all sets is 1:1.

### 2.2. Overall Architecture of Our Model

**Figure 3:**
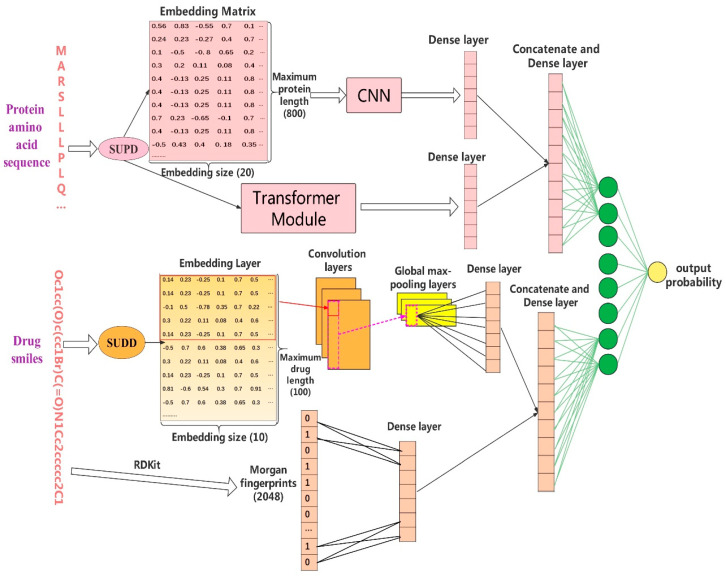
Overall architecture of Multi-TransDTI.

In this work, we propose an end-to-end learning model Multi-TransDTI. The goal of our model is to predict potential drug–protein interactions. The input of our model is the amino acid sequence of the protein and SMILES of the drug. The output is an interaction probability value between the input protein entity and drug entity. The overall model architecture is shown in Figure 3 below.

Our model starts with SUPD and SUDD to transform each SMILES and amino acid sequence into their encoded tokens. All proteins are denoted by set P = {p_1_, p_2_, p_3_, **^……^**, p_i_, p_n_} with a size of n, where i is the i-th protein sequence. All drugs are represented as set D = {d_1_, d_2_, d_3_, **^……^**, d_i_, d_k_} with a size of k, where i is the i-th drug SMILES. All DTI data are represented by set S = {<p_1_,d_1_,0>, <p_2_,d_5_,1>, <p_4_,d_3_,1>, ……, <p_i_,d_i_,0>}, in which each triplet is either a positive or negative sample, and the amount of triplets is the total number for S. More specifically, for each input drug–target pair in S, we first transform the corresponding sequence of p_i_ and d_i_ into encoded tokens $V^{\mathrm{pi}}\in {\mathbb{R}}^{m}$ and $V^{\mathrm{di}}\in {\mathbb{R}}^{v}$, respectively, based on SUPD and SUDD, where m is the dimension of $V^{\mathrm{pi}}$ and v is the dimension of $V^{\mathrm{di}}$. With experiments and tabular statistics in Appendix A, the length of the maximum protein sequence was ultimately set to m = 800, with v = 100 for the maximum drug sequence. The changed S is denoted by S = {<$V^{p1},V^{d1},0$>, <$V^{p2},V^{d5},1$>, <$V^{p4},V^{d3},1$>,……, <$V^{pi},V^{di},0$>}. Next, for newly encoded drug–target token pairs, we flow $V^{pi}$ to both the embedding layer and Transformer module, while $V^{di}$ is sent to embedding layer. The embedding layer is a lookup table of embedding vectors [5,21] in which embedding vector values are trainable and optimized from loss during training. We initialize their values in the form of ‘glorot normal’ [21,36] in tensorflow of our model. Then, we obtain two matrices $M^{V^{pi}}\in {\mathbb{R}}^{m\times u}$ and $M^{V^{di}}\in {\mathbb{R}}^{v\times j}$, where u/j is the embedding size of each token in $V^{pi}$/$V^{di}$. Next, we conduct convolution operations [28] on embedding matrices $M^{V^{pi}}$ along encoded protein tokens and $M^{V^{di}}$ along encoded drug tokens in a 1D fashion to fully extract feature information for both proteins and drugs. After that, we execute global max pooling [37] to filter out the local important residues of encoded proteins and drugs. Eventually, the extracted crucial features are concatenated together to make the final prediction.

### 2.3. Feature of Protein Amino Acid Sequence

#### 2.3.1. Simple Universal Protein Embedding Dictionary (SUPD)

The first step is to generate the embedding dictionary that encodes proteins, noting that protein sequence is composed of capital letters from A to Z [21]. As a result, we generated 18728 possible encoding subsequences. The calculation formula is as follows: 26+26×26+26×26×26=18728. Here, we only calculated up to third-order continuous subsequences instead of fourth-order for the following two reasons [5]: First of all, the fourth-order subsequence would generate approximately 50 thousand potential protein subsequences, which would not only hugely increase the size of the protein dictionary but also amplify the encoding complexity for proteins [33]. Secondly, the third-order subsequence is capable enough of compressing at least half the length of protein sequences and is conducive to feature extraction [38].

The second step is to screen valuable subsequences from all the second-order and third-order subsequences generated in the first step. There is no need to screen first-order subsequences here mainly because after the protein sequence is encoded by second-order and third-order subsequences, the remaining part has to be some single amino acid residues, where each first-order subsequence could play its important role. During the screen, we mainly remove unimportant subsequences according to the frequency of these subsequences in all protein sequences of our BindingDB dataset. As long as the number of second-order or third-order subsequences in one protein sequence is greater than or equal to 7, we then regard the subsequence as a valuable subsequence. Ultimately, we obtained 26 first-order subsequences, 340 second-order subsequences, 108 third-order subsequences, and a simple universal protein embedding dictionary with a length of 474. To the best of our knowledge, this is the first time this method has been implemented to encode protein sequences. By SUPD, not only can we compress the dimension of the embedding matrix and greatly improve the efficiency, but also comprehensively take into account different amino acid residues.

#### 2.3.2. Different Inputs to CNN and Transformer Module

For the CNN module, we first encode the protein sequence according to the SUPD, then generate its corresponding embedding vector for the encoded tokens. The dimension of each embedding vector was experimentally set to 20, but it is a variable parameter. Finally, we put all the embedding vectors together to form the embedding matrix of the protein. This embedding matrix will go a through convolution operation for protein feature extraction.

**Figure 4:**
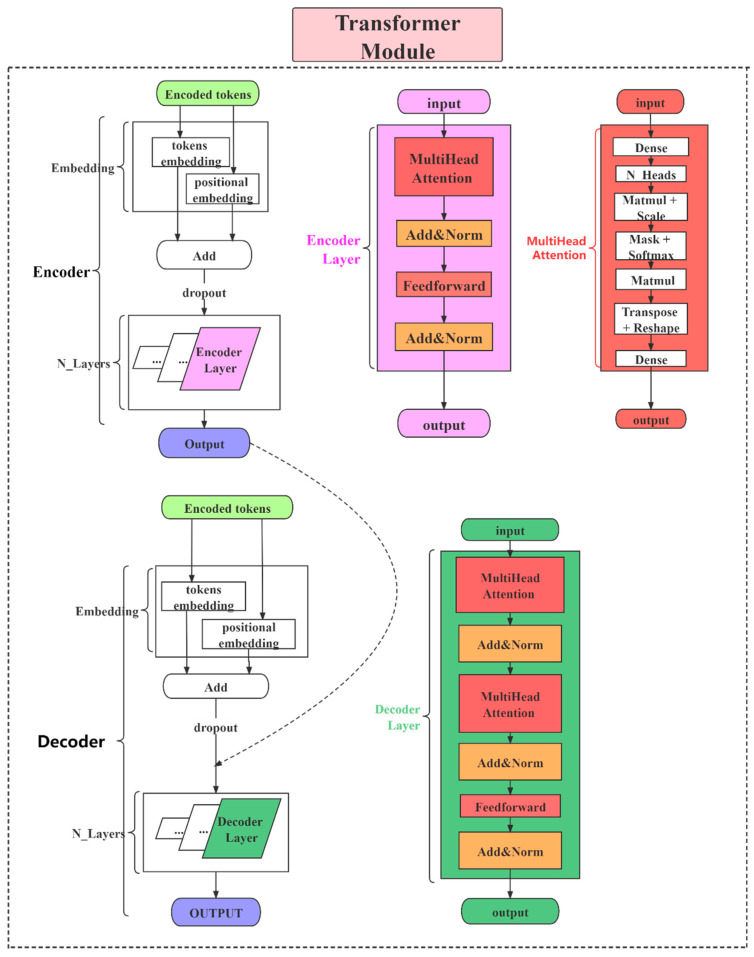
The transformer architecture in our model.

For the transformer module [39], we encode each protein sequence based on SUPD and input the encoded tokens into this module for further protein feature extraction. The specific transformer architecture in our model is shown in Figure 4. We set the N_layers and N_Heads in this module to 4 and 5, respectively. In this part, MultiHead Attention increases the ability of the model to capture different local information and makes the final vector information wider, with the following formulas:

(1) $$Attention\left(Q,k,V\right)=softmax\left(\frac{QK^{T}}{\sqrt{d_{k}}}\right)V$$

$$MultiHead\left(Q,K,V\right)=Concat\left(head_{1},\ldots ,head_{n}\right)W^{o}$$

(2) $$where\;head_{i}=Attention\left(QW_{i}^{Q},KW_{i}^{K},VW_{i}^{V}\right)$$

The feed forward part adopts the dense layer and ReLU function, which can be expressed as

(3) $$FFN\left(x\right)=Relu\left(xW_{1}+b_{1}\right)W_{2}+b_{2\;\;\;\;\;\;\;\;\;}$$

### 2.4. Feature of Drug SMILES

#### 2.4.1. Simple Universal Drug Embedding Dictionary (SUDD)

As one of the most popular drug representations, SMILES (simplified molecular input line entry system) [40] describes a three-dimensional chemical structure with a string of characters, which transforms the chemical structure into a spanning tree and adopts the vertical first traversal algorithm. It is often used as an input to predict potential drug–protein interactions. Some common methods of obtaining drug features are to generate different fingerprints such as Morgan fingerprint [21], graph structure information [41], and so on based on SMILES. The disadvantages of these methods are as follows: Firstly, these features can be regarded as secondary features because they are generated based on SMILES, in which the generation process further depends on extra complicated algorithms [42]. Thus, it increases the complexity of the whole experiment. Secondly, features such as one-hot vectors are generally high dimensional. Although there are some existing dimensionality reduction methods [21], they can add some unavoidable losses of original drug information, as well as increasing the redundancy of the experiment [43]. Consequently, it is rather critical to take SMILES as the original drug indication and extract valuable information to the largest extent. SMILES has different characteristics from protein sequences, mainly consisting of three representation types: symbols, such as @, #; Roman numerals, such as 1, 2; and atoms, such as C, O, and so on. The meaning of these representations and their importance in the whole string could not be analogized with the role of a single amino acid in the whole protein sequence. Therefore, it is inappropriate to generate subsequences on the basis of frequency such as for proteins [44]. In view of the above circumstances, we propose the Simple Universal Drug Dictionary (SUDD) for drug embedding. More specifically, we count the single character in all SMILES strings in the training, validation, and test sets, where we remove the duplicate ones and generate a unique embedding dictionary. To the best of our knowledge, this is the first time this method has been implemented to directly represent drugs with SMILES, which eliminates complexity and redundancy during the feature generation process and achieves promising performance. Ultimately, we obtained a unique drug embedding dictionary with a length of 41.

By SUDD, we encode drug SMILES into the corresponding embedding matrix. For a drug, we first encode each character in drug SMILES according to SUDD, then generate the embedding vectors for each character. The dimension of each embedding is a variable parameter but experimentally set to 10. Finally, we put these vectors together to form the embedding matrix of the drug.

#### 2.4.2. Morgan Fingerprints of Drugs

For each drug, we also used RDKit to generate the Morgan fingerprint with a radius of 2 based on drug SMILES [26]. Thus, each drug can be structurally represented as a binary vector with a length of 2048, where each dimension indicates the existence of specific substructures.

### 2.5. Feature Learning Process of Our Deep Neural Network Model for Both Proteins and Drugs

In our whole model, we learn important local information from protein sequences via CNN and the transformer module [39]. At the same time, we learn drug information via CNN and dense layers. After processing both protein and drug layers, we concatenate these layers and construct the dense layer, resulting in the final output. In order to increase the adaptability and flexibility of the model, nonlinear factors are implemented into each layer. More specifically, each layer is activated by either rectified linear unit (*RELU*) or Sigmoid functions, with Formulas (4) and (5), respectively. In particular, the final output layer is activated by a Sigmoid function with only one unit for classification. The whole neural network model is implemented with Tensorflow (Section 2.4.1).

(4) RELU function:  f(x)=max(0,x)

(5) $$Sigmoid\;function:\;\;f\left(x\right)=\frac{1}{1+e^{-x}}$$

By constructing this deep neural network model, the protein sequence and drug SMILES flow to the final output layer in a feed-forward fashion. We calculate loss with binary cross-entropy. The loss function is as follows:

(6) $$loss\;function:\;\;\;J\left(W,b\right)=-\frac{1}{n}\overset{n}{\underset{i}{\sum }}\left[y_{i}log{\hat{y}}_{i}+\left(1-y_{i}\right)log\left(1-{\hat{y}}_{i}\right)\right]\;\;$$

For neural network techniques, overfitting is a daily common obstacle for most models [45]. Currently, there are several ways proposed to tackle this difficulty, such as regularizing neural networks, where dropout and batch normalization are credited by the majority of scholars. Dropout masks a certain proportion of nodes during training phases, which makes them unavailable to predict results for training labels [46]. Encouraged by that, we implement spatial dropout 1D on the embedding layer.

Finally, we updated the weights using the Adam optimizer with a penalized loss to give a generalized prediction for our model. The penalized function is as follows:

(7) $$L2\;regularization:\;\;\;J_{l2}\left(W,b\right)=J\left(W,b\right)+\lambda \overset{n}{\underset{i}{\sum }}w_{i}{}^{2}$$

## 3. Results 

In our work, we introduce Multi-TransDTI, an end-to-end learning model based on the transformer and newly encoded dictionaries. Not only do we avoid the complex feature generation process, but also hugely reduce the dimensionality of embedding methods for both drugs and proteins without losing information. In addition, we use a multi-view strategy and transformer module to further extract local important residues for proteins while focusing on the global structure, demonstrating promising prospects. Finally, we conducted comprehensive comparison experiments on the BindingDB dataset and evaluated the performance of different state-of-the-art models. Results show that Multi-TransDTI is very competitive in predicting potential DTIs, while maintaining the leading prediction capability on small sample datasets. 

### 3.1. Evaluation Indicators

Selection of threshold: We take AUC as the most important indicator to comprehensively measure the advantages and disadvantages of different models. AUC curve is a monotonic increasing function which represents the dynamic classification capability of the model under a series of thresholds. The optimal threshold comes from the point where the distance between the ordinate and abscissa value on the AUC curve is the maximum. Consequently, we define its calculation formula as follows:

(8) $$Opt\_threshold=\underset{i\in L}{\max}\left(TPR^{i}-FPR^{i}\right)$$

where L is the threshold list and $TPR^{i}\;\mathrm{and}\;FPR^{i}$ are values at the i-th threshold. 

AUC: Area Under Curve. The curve refers to the receiver operating characteristic curve. A series of threshold points drawn by the abscissa value of False Positive Rates (FPR) and ordinate value of True Positive Rates (TPR) are connected together to form the AUC curve. The area under the curve represents its value. The calculation formulas of FPR and TPR are as follows:

(9) $$FPR=\frac{FP}{FP+TN}$$

(10) $$TPR=\frac{TP}{TP+FN}$$

AUPR: Area Under Precision-Recall. The drawing process is rather similar to the AUC curve where the only differences lie in the meaning of abscissa and ordinate. The final value of the AUPR curve is also obtained by calculating the area. The calculation formulas of Recall and Precision are as follows:

(11) $$Recall=\frac{TP}{TP+FN}$$

(12) $$Precision=\frac{TP}{TP+FP}$$

ACC: Accuracy. The calculation formula is as follows:

(13) $$ACC=\frac{TP+TN}{TP+TN+FP+FN}\;$$

F1 score: A trade-off indicator between precision and recall values of the model which represents model stability. The calculation formula is as follows:

(14) $$F1=\frac{2\cdot precision\cdot recall}{precision+recall}$$

### 3.2. Baseline Methods

DNN: we combined Morgan fingerprints with 100-dimensional encoded tokens based on SUDD as drug feature input, and 800-dimensional encoded tokens based on SUPD as protein features. Then, we flowed them to four layers of the deep neural network, with hidden sizes of 128 and 32 to complete the drug–target interaction prediction task.

Model-CPI: the paper [41] proposes an end-to-end representation learning for compounds and proteins, developing a new CPI prediction by combing a graph neural network (GNN) for compounds and a convolutional neural network (CNN) for proteins. We used the same parameters in the source code, without making any changes.

Moltrans: the paper [5] applies an augmented transformer to extract and capture the semantic relationships among substructures generated from massive unlabeled biomedical data. It conducts extensive experiments on different datasets and achieves promising performance. We employed the same parameter settings from the paper.

DeepConv-DTI: as one of the state-of-the-art models in DTI binary prediction tasks, the paper [21] implements CNN, global max pooling and batch normalization layers to extract local patterns in protein sequences, using dense layers on Morgan fingerprints of the drugs. We used all the same optimal hyperparameters described in that paper.

### 3.3. Comparisons of Different Models

To evaluate the competitiveness of our model, we conducted comparative experiments with state-of-art models proposed previously for drug–target interaction prediction. Our model generally outperforms state-of-the-art models in datasets of different proportions. 

**Figure 5:**
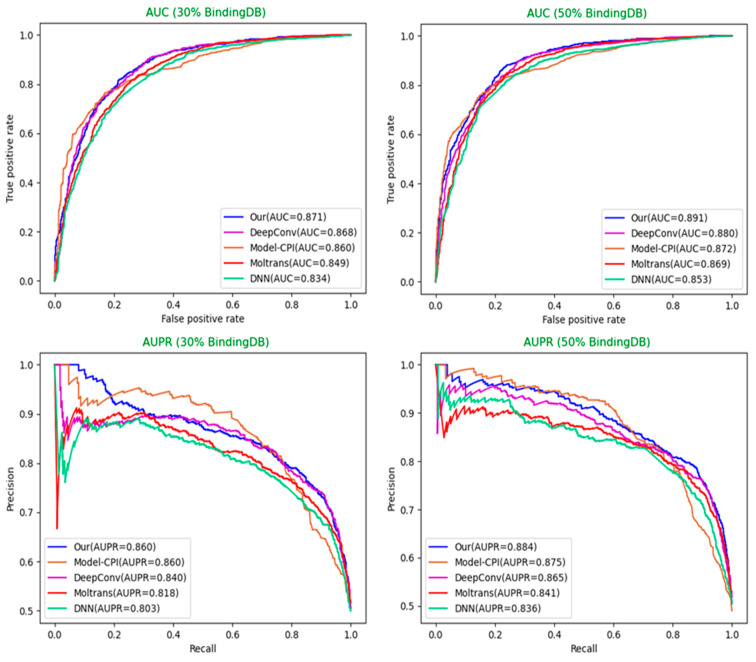
Model comparisons of AUC and AUPR on 30% and 50% BindingDB dataset (Our = MultiTrans-DTI).

**Figure 6:**
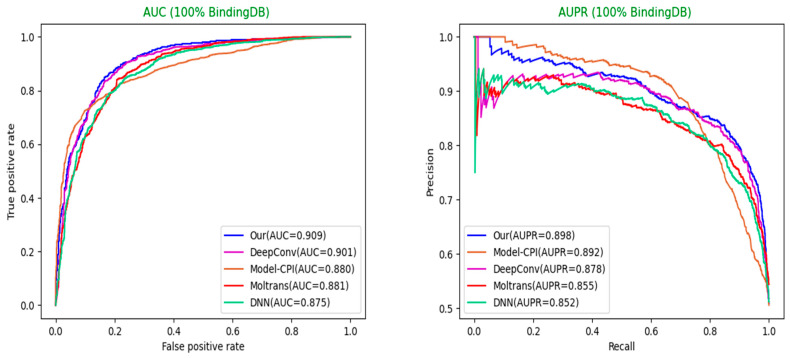
Our model achieves the best AUC and AUPR on 100% BindingDB dataset (Our = MultiTransDTI).

Selection of different optimal models: For each model, we used the optimal hyperparameters either given in the source codes or described in the paper. Moreover, each model sets sufficient epochs until the loss value converges completely, so that the weights of the model reach optimality. After that, we repeated the training, validation, and test process on each model three times and adopted the best model among them. The optimal model weights of different models are saved based on the AUC values in the validation set. Finally, we used weights saved to perform the predictions on the test set for each model and calculated a series of indicator values. The comparison results of different models in various indicators are shown in Table 3, Table 4 and Table 5 below. The comparison diagrams for AUC and AUPR are shown in Figure 5 and Figure 6.

**Figure 7:**
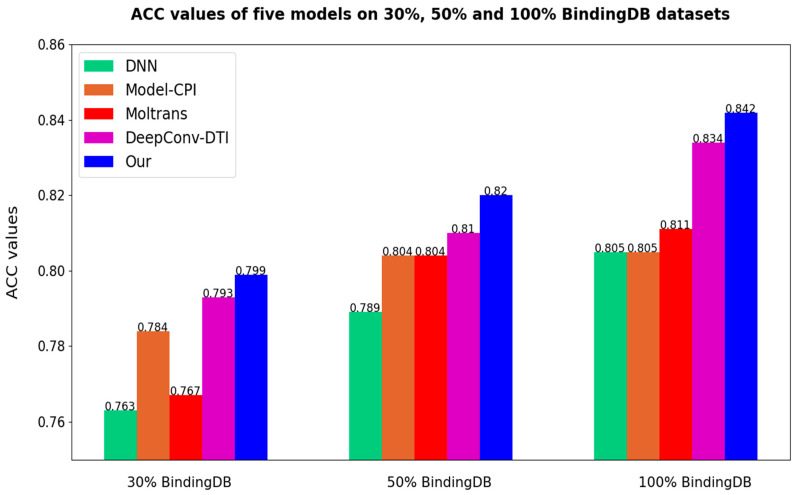
Comparisons of different models on ACC (Our = Multi-TransDTI).

**Figure 8:**
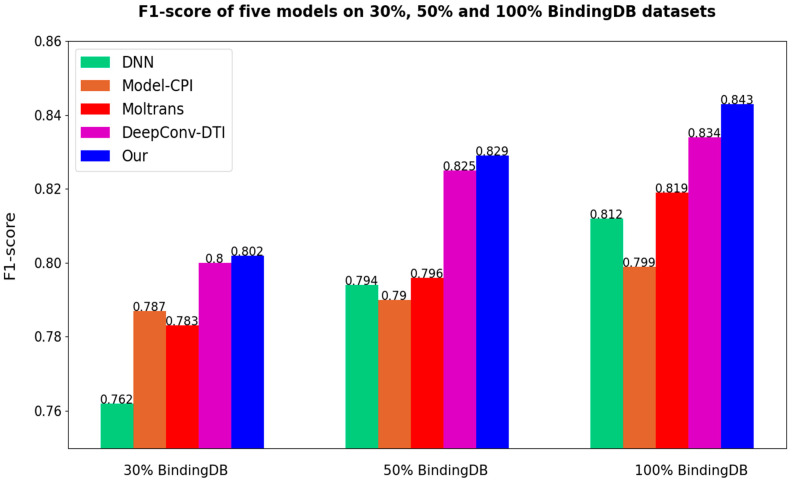
Comparisons of different models on F1-socre (Our = Multi-TransDTI).

After all the models are set to the optimal threshold, the bar charts of ACC and F1-score of different models are as follows in Figure 7 and Figure 8. As the results prove once again, our method is highly competitive.

### 3.4. Ablation Experiments

In the end, we also compared different channels of our model to observe the contribution of different channels and the stability of model performance. For drugs and proteins, we separately measured the operation importance of Morgan fingerprints, drug_CNN, protein_CNN, and protein_transformer. Among them, protein_CNN represents proteins that are processed merely by the CNN channel while keeping two drug channels constant. The same is true for the other three channels. The ALL channel denotes the integrated model without removing any parts or channels. We carried out each experiment three times and selected an optimal value based on the AUC value. The specific results are shown in Table 6. Among them, AUC and AUPR show a tiny difference, which means higher AUC or AUPR channels have relatively more than one optimal threshold. On the other hand, all channels display very competitive performance in ACC and F1-score under the best threshold. Another merit observed in Table 6 is that the use of a multi-view strategy and multiple information features could make the model more stable and improve the performance in every aspect.

## 4. Discussion

The biological process of targets (proteins) in our body uniquely influences the fundamental way of life every day, in which their inhibition and activation are highly related to drug interactions. In consequence, predicting potential drug–target interactions has great value in drug repositioning, discovery, etc. Given that biological assays for identifying drug candidates are time consuming and labor intensive, computational prediction approaches have been introduced. However, existing methods generally extract important local residues of protein sequences through CNN-based models, ignoring their global structure and generating an embedding matrix of high dimension. At the same time, the feature generation of drugs (SMILES, InchI, and so on) relies heavily on complex libraries, such as RDKit for fingerprints, resulting in the unavailability of some drug features. Furthermore, the weak generalization ability of different models has become a common problem due to the unicity of features and insufficient learning in the case of small sample data. In view of all the above concerns, we implement a transformer, simple universal dictionaries, and a multi-view strategy, respectively, achieving highly competitive performance in experiments.

## Figures and Tables

**Table 1:** BindingDB dataset.

Name	Positive Samples	Negative Samples	Total Samples	Number of Drugs	Number of Proteins
BindingDB (100%)	6571	6571	13,142	7137	1253

**Table 2:** BindingDB datasets of different proportions.

Percent	Train/Valid/Test	Ratio of Positive and Negative Samples in Train/Valid/Test
100%	9200/1970/1972	1:1/1:1/1:1
50%	4600/1970/1972	1:1/1:1/1:1
30%	2770/1970/1972	1:1/1:1/1:1

**Table 3:** Comprehensive performance of different models on 100% BindingDB.

Methods	AUC	AUPR	ACC	F1-Score	Threshold
DNN	0.875	0.852	0.805	0.812	0.351
ModelCPI	0.880	0.892	0.805	0.799	0.654
Moltrans	0.881	0.855	0.811	0.819	0.514
DeepConv	0.901	0.878	0.834	0.834	0.552
Multi-TransDTI	0.909	0.898	0.842	0.843	0.604

**Table 4:** Comprehensive performance of different models on 50% BindingDB.

Methods	AUC	AUPR	ACC	F1-Score	Threshold
DNN	0.853	0.836	0.789	0.794	0.521
ModelCPI	0.872	0.875	0.804	0.790	0.496
Moltrans	0.869	0.841	0.804	0.796	0.349
DeepConv	0.880	0.865	0.810	0.825	0.316
Multi-TransDTI	0.891	0.884	0.820	0.829	0.397

**Table 5:** Comprehensive performance of different models on 30% BindingDB.

Methods	AUC	AUPR	ACC	F1-Score	Threshold
DNN	0.834	0.803	0.763	0.762	0.489
ModelCPI	0.860	0.860	0.784	0.787	0.387
Moltrans	0.849	0.818	0.767	0.783	0.364
DeepConv	0.868	0.840	0.793	0.800	0.355
Multi-TransDTI	0.871	0.860	0.799	0.802	0.553

**Table 6:** Ablation experiments on 100% BindingDB dataset.

Channels	AUC	AUPR	F1-Score	ACC
Protein_CNN	0.905	0.893	0.836	0.836
Protein_transformer	0.893	0.878	0.838	0.830
Drug_CNN	0.896	0.888	0.836	0.829
Drug_fingerprints	0.905	0.894	0.837	0.833
ALL	0.909	0.898	0.842	0.843

## Data Availability

Publicly available datasets were analyzed in this study. This data can be found here: https://github.com/nick1997a/model, (accessed on 26 February 2022) [47].

## Appendix A

### Appendix A.1. Maximum Embedding Length of Proteins

We calculated the information loss rate of amino acid sequences under different maximum protein embedding lengths for the 100% BindingDB dataset, finally determining 800 as the optimal value. The specific ratios are shown in Table A1.

**Table A1:** Protein maximum length coverage.

Maximum Embedding Length of Protein	Coverage on Training Set	Coverage on Validation Set	Coverage on Test Set	Coverage on All Sets
600	85.8%	84.9%	85.0%	85.5%
700	92.5%	92.8%	91.9%	92.4%
800	96.2%	96.4%	96.1%	96.2%

### Appendix A.2. Maximum Embedding Length of Drugs

We implemented same process for drug SMILES. The specific ratios are shown in Table A2.

**Table A2:** Drug maximum length coverage.

Maximum Embedding Length of Drug	Coverage on Training Set	Coverage on Validation Set	Coverage on Test Set	Coverage on All Sets
80	87.3%	88.5%	88.8%	87.7%
90	91.7%	92.8%	92.1%	91.9%
100	93.0%	93.8%	92.8%	93.1%

### Appendix A.3. Hyperparameter Setup of Our Model

After the framework of our model became fixed, we conducted some hyperparameter experiments. Table A3 shows some tested and selected values. These parameters vary with the network structure of the model.

**Table A3:** Hyperparameters of our model.

Hyperparameter	Range	Selected Value
Learning rate	[0.01,0.001,0.0001,0.0002]	0.0001
Decay rate	[0.01,0.001,0.0001]	0.0001
Activation function	[Sigmoid, ReLU, ELU]	ReLU, Sigmoid
Dropout rate	[0,0.1,0.2,0.3,0.4,0.5]	0.2
Epoch	0–60	50
Batch size	[8,16,32,64,128]	16,32
